# One month of contemporary dance modulates fractal posture in aging

**DOI:** 10.3389/fnagi.2014.00017

**Published:** 2014-02-25

**Authors:** Olivier A. Coubard, Lena Ferrufino, Tetsushi Nonaka, Oscar Zelada, Blandine Bril, Gilles Dietrich

**Affiliations:** ^1^The Neuropsychological Laboratory, CNS-FedParis, France; ^2^Groupe de Recherche Apprentissage et Contexte, Ecole des Hautes Etudes en Sciences SocialesParis, France; ^3^Research Institute of Health and Welfare, Kibi International UniversityTakahashi, Okayama, Japan; ^4^Facultad de Medicina Dr. Aurelio Melean, Universidad Mayor de San SimonCochabamba, Bolivia; ^5^Techniques et Enjeux du Corps, Université Paris DescartesParis, France

**Keywords:** aging, motor control, posture, contemporary dance, fractals, plasticity

## Abstract

Understanding the human aging of postural control and how physical or motor activity improves balance and gait is challenging for both clinicians and researchers. Previous studies have evidenced that physical and sporting activity focusing on cardiovascular and strength conditioning help older adults develop their balance and gait and/or decrease their frequency of falls. Motor activity based on motor-skill learning has also been put forward as an alternative to develop balance and/or prevent falls in aging. Specifically dance has been advocated as a promising program to boost motor control. In this study, we examined the effects of contemporary dance (CD) on postural control of older adults. Upright stance posturography was performed in 38 participants aged 54–89 years before and after the intervention period, during which one half of the randomly assigned participants was trained to CD and the other half was not trained at all (no dance, ND). CD training lasted 4 weeks, 3 times a week. We performed classical statistic scores of postural signal and dynamic analyses, namely signal diffusion analysis (SDA), recurrence quantification analysis (RQA), and detrended fluctuation analysis (DFA). CD modulated postural control in older trainees, as revealed in the eyes closed condition by a decrease in fractal dimension and an increase in DFA alpha component in the mediolateral plane. The ND group showed an increase in length and mean velocity of postural signal, and the eyes open a decrease in RQA maximal diagonal line in the anteroposterior plane and an increase in DFA alpha component in the mediolateral plane. No change was found in SDA in either group. We suggest that such a massed practice of CD reduced the quantity of exchange between the subject and the environment by increasing their postural confidence. Since CD has low-physical but high-motor impact, we conclude that it may be recommended as a useful program to rehabilitate posture in aging.

## Introduction

As the frequency of falls increases with age, developing strategies to reduce the risk of falls in aging is a major issue in public health (Judge, 2003). Indeed falling in older adults has dramatic consequences in terms of morbidity and mortality (Rubenstein, 2006). Besides environmental and hazard management, exercise in aging has been shown to be efficient in reducing the frequency of falls (Day et al., 2002). As regards the various types of exercise, some studies have pointed out the need to distinguish between physical- vs. motor-dominant activities (e.g., Voelcker-Rehage et al., 2010). Physical-dominant fitness is based on cardiovascular and strength conditioning in activities such as walking, running or cycling, with beneficial impacts on physical and cognitive health (for reviews see Hillman et al., 2008; Voss et al., 2011). Motor-dominant fitness stands for the motor learning of new skills such as balance, fine motor coordination, motor flexibility, and motor speed in activities as dance, gymnastics, or martial arts (Voelcker-Rehage et al., 2010). In the last decade, dance has been put forward as a promising program to develop balance and prevent falls in older adults (American Geriatrics Society, 2001; Judge, 2003).

Preliminary reports without control groups have suggested that dance may improve balance in aging. Hamburg and Clair (2003) observed in 36 older adults aged 63–86 years that a 7-week Motivating Moves program based on Laban movement increased their balance in a modified time up-and-go and standing toe/heels raises, as well as their gait cadence and velocity. Alpert et al. (2009) reported an increasing trend on the Equitest Sensory Organization Test in 13 women aged 52–88 undergoing jazz dance for 15 weeks. Krampe et al. (2010) observed in 11 older adults an increasing trend in time up-and-go and functional reach after a 6-week dance intervention based on the Lebed method.

Observational studies with control groups and randomized controlled trials have corroborated the beneficial impact of dance on balance in aging. Shigematsu et al. (2002) showed that 20 women aged 72–87 years, compared to 18 untrained participants, had better balance in the single-leg stance with their eyes closed and in functional reach, as well as higher walking agility in walking time around two cones after 12 weeks (3 sessions a week) of dance-based aerobic exercise. Hui et al. (2009) reported that 52 older adults aged 68.0(Mean) ± 4.5(*SD*) years trained to 12 weeks (2 sessions a week) of low-impact aerobic dance, compared to 42 untrained controls, improved not their static balance in the Physical Performance Battery, but their dynamic balance and mobility in time up-and-go. Sofianidis et al. (2009) showed in 14 older adults aged 69.2 ± 4.3 years, compared to 12 untrained participants, that 10 weeks (2 sessions a week) of traditional Greek dance improved their static balance by decreasing the center-of-pressure (CoP) displacements and trunk sways in one-leg stance, as well as their dynamic balance by increasing the range of trunk rotation during weight shifting in both mediolateral or anteroposterior planes.

More recently, Granacher et al. (2012) observed that 8 weeks (2 sessions a week) of salsa dance in 14 older adults aged 63–82 years, compared to 14 untrained controls, merely *tended* to improve their static balance in the single-leg stance on a force platform, whereas dynamic balance in walking on an instrumental walkway was *significantly* enhanced, as evidenced by higher stride velocity and strength vs. lower stride time. Consistently Kattenstroth et al. (2013) showed that 24 weeks (1 session a week) of a dance program developed for elderly people improved not static but dynamic balance in 25 older adults aged 68.6 ± 1.4 years, compared to 10 untrained participants, as assessed in different conditions on a force platform. Finally, Krampe (2013) observed that only 6 weeks (3 sessions a week) of a dance program based on the Lebed method yielded a mild to moderate effectiveness in several measures of balance (multidirectional reach) and mobility (velocity, step length, and functional ambulation profile) in 13 older adults aged 64–96 years, by comparison to 11 untrained controls.

As a logical consequence of dance benefits on balance, cross-sectional studies have confirmed that older social dancers have better balance, posture, and gait (Verghese, 2006; Zhang et al., 2008; Kattenstroth et al., 2010), a more stable walking pattern (Verghese, 2006), and faster leg reaction time (Zhang et al., 2008) than older non-dancers.

Balance comprises a blend of multisensory integration of visual, vestibular, proprioceptive, and exteroceptive afferences, central motor processing, and adapted response generation (Nashner, 1976). As visual perception is impossible without movement (Martinez-Conde et al., 2013), balance is primarily action for building perception through movement so as to explore the world with the body. Over the last 20 years, a transdisciplinary approach has favored the emergence of new theoretical frameworks and novel tools to examine non-linear dynamic systems or physiological rhythms, such as postural signal. Such an approach has provided insight into the motor control underpinnings, and helped researchers understand further how human balance may be optimized by a specific training.

The goal of this study was to examine the effects of 1-month practice of contemporary dance (CD) on postural control of older adults, as compared to older adults who were not trained at all (ND). Using a force platform, we measured the CoP displacements in upright stance before and after the intervention. We calculated classical statistic scores (length, surface, velocity), as well as the Romberg quotient to assess the effect of vision (Van Parys and Njiokiktjien, 1976), and fractal dimension to quantify the exchange between the subject and the environment (Hausdorff, 2007). Additionally, we examined the dynamics of CoP displacements using dynamic frameworks: signal diffusion analysis (SDA) (Collins and De Luca, 1993), recurrence quantification analysis (RQA) (Riley et al., 1999), and detrended fluctuation analysis (DFA) (Peng et al., 1994).

In SDA, the CoP trajectories can be modeled as fractional Brownian motion that has at least two control mechanisms: open- and closed-loop control schemes over short- and long-term intervals, respectively. The stabilogram diffusion plot, in which the trajectory of the mean square displacement is plotted as a function of time interval, changes slope after a critical point thus exhibiting short- and long-term regions (Collins and De Luca, 1993). Normal aging is associated with motor stiffness, which yields higher critical mean square displacement and higher critical time interval in SDA (Collins et al., 1995).

RQA is a non-linear method that plots the local recurrence of data points in a reconstructed phase space and provides, among other measures, the degree of autocorrelation, given by recurrence (%REC) and determinism (%DET) on the one hand, and mathematical stability, given by maximal line diagonal (MAXL) on the other. RQA is a useful alternative to SDA for periodic data, but its measures cannot be taken as absolute but relative to a manipulated variable (Riley et al., 1999). In brief, %REC, %DET, and MAXL decrease with increasing behavioral flexibility (Webber and Zbilut, 2005), and thus increase with increasing motor stiffness as in Parkinson's disease (Schmit et al., 2006).

DFA allows the detection of fractal scaling in non-stationary time series (Peng et al., 1994, 1995). In this method, the time series of CoP displacements (i.e., Euclidean distances) to be analyzed (with *N* samples) is integrated first. Then, the integrated time series is divided into boxes of equal length, *n*. In each box, a least squares line is fit to the data representing the trend in this box. The *y* coordinate of the straight line segments is denoted by *y_n_*(*k*). Next, the integrated time series, *y*(*k*), is detrended by subtracting the local trend, *y_n_*(*k*), in each box. The root mean square fluctuation of this integrated and detrended time series is given by equation (1).

(1) $$F(n)=\sqrt{\frac{1}{N}\sum_{k=1}^{N}{\left[y(k)-y_{n}(k)\right]}^{2}}$$

This computation is repeated over all time scales (box sizes) to characterize the relationship between the average function *F*(*n*) and the box size *n*. Typically, *F*(*n*) increases with box size. A linear relationship on a log–log indicates the presence of power law (fractal) scaling. Under such conditions, the fluctuations can be characterized by a scaling exponent α, the slope of the line relating log *F*(*n*) to log *n*.

In a previous study (Ferrufino et al., 2011), we examined the effects of two types of motor training, CD and fall prevention, on postural control in older adults. We showed that a 4.4-month practice of CD enhanced the critical time interval in SDA and reduced %REC and MAXL in RQA, suggesting higher motor flexibility after CD, as compared to fall prevention which yielded a reverse tendency. In our previous study (Ferrufino et al., 2011), the training was distributed over time: 1 h a week for a 4.4 month duration. In the present study, we examined whether a massed CD training—namely 4.5 h a week for 1-month in duration—might induce similar or different effects in nature and extent on postural control of older adults. We performed an extensive exploration of classical and dynamic postural measures as the effects of such massed CD training were expected to be subtle.

## Methods

### Participants

Thirty-eight Bolivian natives participated in the study, which was approved by the local ethics committee (Universidad Abierta para Adultos Mayores UNI-3, Cochabamba). Instructions were in Spanish, which was the first language of all participants. They were right-handed, had normal or corrected-to-normal vision, no neurological disorders, and were unaware of the goal of the experiment.

Table 1 provides the participants' sex, age, body mass index (BMI), defined as the weight divided by the squared height in kg.m^−2^, years of education, socio-cultural level defined as the years of education weighted by socio-professional experience (Kalafat et al., 2003), and their score in the Mini-Mental State Examination (MMSE) for cognitive status (Folstein et al., 1975).

**Table 1:** **Number (gender) or Mean ± Standard Deviation (age, BMI, education, socio-cultural level, MMSE) for the groups of participants (CD, contemporary dance; ND, no dance)**.

	***CD***	***ND***
Gender (women/men)	19/0	18/1
Age (years)	70.6±7.3	72.6±8.6
BMI (kg.m^−2^)	26.5±3.8	27.0±3.9
Education (years)	13.4±3.7	9.9±3.6
Socio-cultural level (/4)	3.6±0.8	3.0±1.1
MMSE (/30)	27.7±1.6	26.7±1.9

Nineteen participants aged 59–81 years made up the CD group, and 19 participants aged 54–89 years made up the ND group. The two groups were matched in gender (χ^2^ < 1), age (*t* < 1), BMI (*t* < 1), socio-cultural level [*t*_(36)_ = 1.93, *P* > 0.05], their score in the MMSE [*t*_(36)_= 1.84, *P* > 0.05], but not in years of education, which was higher in the CD group [*t*_(36)_= 2.87, *P* < 0.01].

The CD and ND were matched in past physical activity as measured by the years of practice in each of the following activities. Twenty-four of them had done 4.5 ± 4.4 years of gymnastics in the past, 12 of them had done 1.4 ± 3.4 years of yoga, 6 of them had done 2.7 ± 3.4 years of Bolivian traditional dance (e.g., Caporales, Kantus, Llamerada, Potolo, Suri Sikuris, Tobas), 4 of them had done 1.4 ± 0.8 years of CD, and 1 had done Tai Chi Chuan for 1 year. For each activity, the difference between the two groups failed to reach statistical significance (Mann–Whitney, *P* > 0.05).

### Apparatus

We examined static posture using a Techno-Concept platform (Céreste, France), which consisted of two dynamometric clogs, one for each foot, embedded in a board so that the angle made by the feet was 30°. The displacements of the CoP were recorded for 51.2 s and digitized at 40 Hz using a 16 bit analogical-digital converter.

### Training program

Participants were recruited from their leisure clubs and randomly assigned to either the CD or the ND group. The training was conducted by a professional instructor and supervised by another professional instructor. The training program lasted 4 weeks, the frequency was three times a week (on Monday, Wednesday, and Friday), and each session lasted 1.5 h (from 3:00 to 4:30 p.m.). Music could be used during up to 25% of the session duration. In the ND group, participants did not practice any physical or motor training during the intervention period.

#### Contemporary dance

Four stages made up the training session: opening, warm-up, improvisation, and closure. (1) Opening consisted in some variations of the action of walking and a free dance on a popular music. (2) Warm-up. In this stage, the body was prepared to dance by passive and active movements of joints, movements of muscular stretching, coordinated breath and movement, and body positioning and alignment. (3). Improvisation. This stage was the core of CD training. Given a constraint (action, idea, location, music, object, or word), the stage was organized around four steps: (i) individual exploration of the constraint; (ii) exploration in pairs or more taking into account the other trainees; (iii) each duo or group presented the work developed in (i) and (ii); (iv) solo improvisation and development of a natural movement to express one's own sensations. Such an organization allowed participants to work through two types of improvisation: *solo improvisation* or *improvisation for oneself* in steps (i) and (iv), and *framed improvisation* or *reflexive availability* in steps (ii) and (iii), which were favored at all steps by dance tools suggested by the instructor (Ferrufino and Coubard, 2011). (4). Closure. The body was cooled down by breath and massages. For each session, 10, 25, 45, and 10 min were, respectively, dedicated to stages 1–4. Participants did not take part in other physical or motor training programs during the intervention period.

### Postural recording

Participants underwent postural recordings before and after the training intervention. In a quiet normally lit room, they stood in an upright posture on the platform, barefoot and with their arms comfortably at their sides. The instruction was to breathe normally and to remain relaxed. The participants were given two conditions. In the eyes closed condition, participants wore a mask in front of their eyes enabling darkness. In the eyes open condition, they fixated a black circular surface at eye level subtending 1.5° of visual angle, at a distance of 150 cm (Coubard, 2011). When necessary, participants wore their usual spectacle correction. The order of conditions was counterbalanced.

### Postural measurements

**Figure 1:**
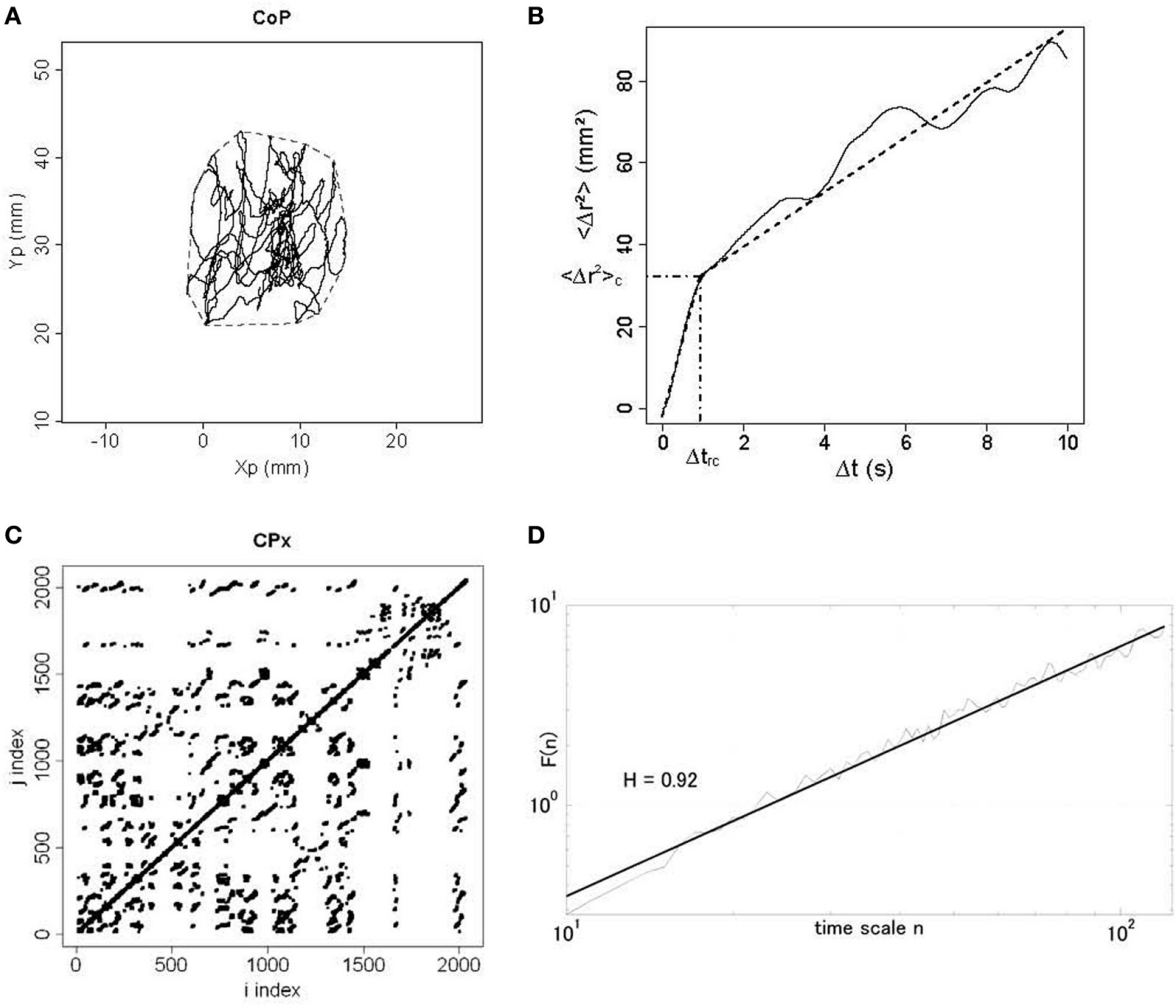
**Different plots from a dancer participant in the eyes open condition in the post-test period. (A)** Statokinesigram of center-of-pressure (CoP) displacements. Anteroposterior movement of CoP (Yp) is plotted against mediolateral movement (Xp) in millimeters (mm). The convex hull area is shown in dotted line. **(B)** Stabilogram-diffusion plot. The mean square CoP movement (<Δ*r*^2^>) in mm^2^ is plotted as a function of time interval (Δ*t*) in seconds. We show experimental data in full line and theoretical fit in dotted line for the first and second regressions. Semi-dotted lines indicate the coordinates of the critical point between the two regressions, namely <Δ*r*^2^>_*c*_ on the *y* axis and Δ*t_rc_* on the *x* axis. **(C)** Recurrence quantification analysis plot. Here we show the phase space for mediolateral movement (CPx) for a radius of 3%. It means that for a 3%-radius sphere of the mean distance between data points, we calculated for *i* = 1-N and *j* = 1-N (where *N* is the total number of data points) the distance between data points *x*(*i*) and *x*(*j*), and darkened every recurrent point for which the distance was below the radius. For *i* = j, the distance is zero resulting in the central diagonal line. **(D)** Detrended fluctuation analysis plot. In a log–log plot, we show the DFA α exponent *F*(*n*) in thin line and the corresponding regression in thick line, as a function of time scale *n*.

Using home-made scripts under Matlab 7.0 (The MathWorks, Natick, MA, USA) and R (www.r-project.org, Institute for Statistics and Mathematics, Vienna, Austria), we processed the raw data provided by the manufacturer software as follows. The first and last of the 2048 samples were discarded as they could exhibit artifacts due to onset and offset of the recording, respectively. Only positions of the CoP in mediolateral (*x*) and anteroposterior (*y*) planes were kept for analysis. An example of the CoP displacements is illustrated in Figure 1A. We then calculated statistic scores and performed dynamic analyses.

#### Statistic scores

We measured the length (in millimeters, mm), the confidence ellipse area and the convex hull area (in mm^2^), which include 90 and 100% of CoP positions respectively (Andrew, 1979) (see Figure 1A), as well as the mean and variance velocities (in mm.s^−1^) of the CoP displacements. We also calculated the Romberg quotient as the confidence ellipse area in the eyes closed condition divided by that in the eyes open condition, and the fractal dimension (FD) ratio given by equation 2 (Chiari et al., 2000).

(2) $$FD=\frac{\log(N)}{\log(N)+\log\left(\frac{\text{RANGE}}{\sum_{i\;=\;1}^{N-1}\Delta r_{i}}\right)}$$

#### Signal diffusion analysis (SDA)

We plotted the planar mean square displacement of the CoP <Δ*r*^2^> in mm^2^ (where *r* is the sum of the displacements in mediolateral and anteroposterior planes, and the brackets means the average over time) as a function of time interval Δ*t* in seconds (see Figure 1B). For the short- and long-term regions, we calculated the diffusion coefficients *D_S_* and *D_L_* in mm^2^.s^−1^ from the slopes of linear-linear plots of <Δ*r*^2^> against Δ*t* curves, and the scaling exponents *H_S_* and *H_L_* from the log–log plots of such curves. The critical point separating the two regions was defined as the critical mean square displacement <Δ*r*^2^>_*c*_ (in mm^2^) on the *y* axis, and the critical time interval Δ*t_rc_* (in seconds) on the *x* axis (see Figure 1B).

#### Recurrence quantification analysis (RQA)

Input parameters were set as follows according to Webber and Zbilut's guidelines (Webber and Zbilut, 2005): time lag was 50 ms corresponding to 2 samples; the embedding dimension was 12; the radius took the values 2–3% of the mean distance between data points as lower or higher percentages led to not enough or too many recurrent data points; finally, we adopted a conservative value, 3, to define a diagonal line segment. As outputs, we measured the percentage of recurrence (%REC) and of determinism (%DET), and the maximal diagonal line (MAXL). These three measures were calculated separately for the *x* and *y* planes of the CoP fluctuations, and for radii of 2–3%. An example of RQA plot is shown in Figure 1C.

#### Detrended fluctuation analysis (DFA)

We computed a time series of CoP displacements between successive samples in the position time series in both mediolateral and anteroposterior planes. DFA α exponents were calculated for sliding windows of 30 s (1200 samples), starting from the beginning of each time series, estimated over the scaling range 10 < *n* < 120 where *n*_max_ ≈ *N*/10 = 120 is the maximal time scale for which the DFA scaling analysis is reliable. The windows were slid by 1 s increments until 21 windows in total were obtained, yielding 21 DFA α exponents for each condition and each plane (mediolateral and anteroposterior). An example of DFA plot is illustrated in Figure 1D.

### Statistical analysis

All measures were submitted to analyses of variance (ANOVAs) with Group (2 levels: CD vs. ND) as between-participant factor, Period (2 levels: pre-test vs. post-test) and Eye (2 levels: eyes closed, eyes open) as within-participant factors. *Post-hoc* tests were calculated using Fisher's Least Significant Difference (LSD) method. Distributional information was given by standard errors (SE). For all analyses, we used Statistica 7.0 (StatSoft, Tulsa, OK, USA).

## Results

### Statistic scores

Results are shown in Table 2. Three-Way ANOVAs with Group, Period, and Eye as factors showed a main effect of Eye for length [*F*_(1, 36)_ = 35.5, *P* < 0.01], confidence ellipse area [*F*_(1, 36_) = 10.7, *P* < 0.01], convex hull area [*F*_(1, 36)_ = 17.4, *P* < 0.01], mean velocity [*F*_(1, 36)_ = 35.5, *P* < 0.01], and variance velocity [*F*_(1, 36)_ = 18.3, *P* < 0.01], with higher values in the eyes closed condition for all measures (see Table 2). For the Romberg quotient, there was a main effect of Period [*F*_(1, 36)_ = 4.28, *P* < 0.05] with higher value before (1.46) than after (1.16) the intervention.

**Table 2:** **Mean ± SE of statistic scores for the groups of participants (CD, contemporary dance; ND, no dance)**.

	**Eyes closed**		**Eyes open**		***F***	***P***
	***CD***	***ND***	***CD***	***ND***		
**LENGTH (mm)**						
Pre-test	644.5±36.7	622.8±40.5	548.1±25.6	519.5±37.1		
Post-test	615.1±29.3	674.7±48.2	541.3±19.1	588.2±33.4	<1	–
**ELLIPSE AREA (mm^2^**)						
Pre-test	195.2±18.7	194.1±21.6	169.5±16.0	138.7±18.7		
Post-test	228.4±32.4	199.2±29.6	203.6±26.4	182.3±27.3	<1	–
**HULL AREA (mm^2^**)						
Pre-test	290.6±26.7	290.5±44.2	234.5±22.3	188.7±21.6		
Post-test	329.7±41.0	326.1±46.6	266.4±29.1	253.8±35.1	<1	–
**MEAN VELOCITY (mm.s^−1^**)						
Pre-test	12.6±0.7	12.2±0.8	10.7±0.5	10.2±0.7		
Post-test	12.0±0.6	13.2±0.9	10.6±0.4	11.5±0.7	<1	–
**VARIANCE VELOCITY (mm.s^−1^**)						
Pre-test	85.3±12.3	82.2±14.2	56.9±7.6	53.2±8.6		
Post-test	81.1±11.2	105.5±17.7	62.6±6.4	69.1±8.9	<1	–
**FRACTAL DIMENSION RATIO**						
Pre-test	1.69±0.03	1.68±0.02	1.66±0.02	1.67±0.03		
Post-test	1.66±0.03*	1.70±0.03	1.65±0.02	1.71±0.02	<1	–
**ROMBERG QUOTIENT**	**EYES CLOSED/EYES OPEN**					
Pre-test	1.32±0.19	1.60±0.21				
Post-test	1.19±0.11	1.14±0.09			1.33	0.257

ANOVAs' F- and P-values are those of the third-order interaction (except for the Romberg quotient for which F- and P-values are those of the second-order interaction). Asterisks only indicate statistical significant difference (LSD, P < 0.05) between pre- and post-test periods.

Critical for our hypothesis, the Group*Period interaction was significant for length [*F*_(1, 36)_ = 5.93, *P* < 0.05], mean velocity [*F*_(1, 36)_ = 5.94, *P* < 0.05], and fractal dimension [*F*_(1, 36)_ = 5.63, *P* < 0.05]. This interaction was due to higher values in the post- than in the pre-test period for the ND group for the length (631 > 571 mm; LSD, *P* < 0.05) and mean velocity (12.3 > 11.2 mm.s^−1^; LSD, *P* < 0.05). For fractal dimension, the interaction was due to a decrease between the two periods in the CD group (1.67 > 1.65), whereas a reverse tendency was observed in the ND group (1.68 < 1.70), though *post-hoc* failed to reach significance.

**Figure 2:**
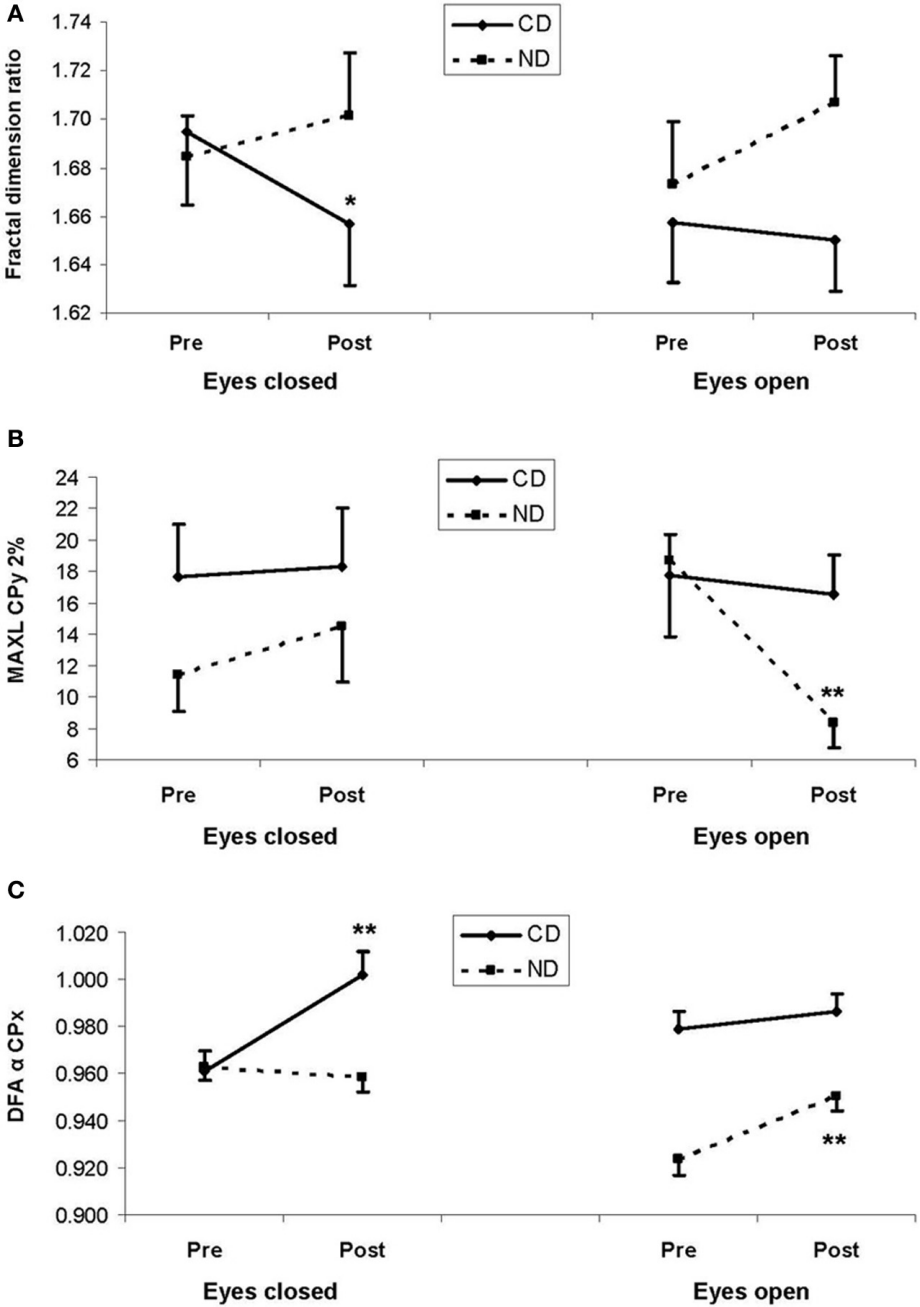
**(A)** Mean fractal dimension ratio, **(B)** mean maximal diagonal line (MAXL) for mediolateral movement (CPx) for a 2%-radius from the recurrence quantification analysis, and **(C)** mean DFA α exponent for mediolateral movement (CPx) from the detrended fluctuation analysis. In the three plots, results are shown as a function of pre-test (Pre) and post-test (Post) periods, and of eyes closed (left panels) and eyes open (right panels) conditions, for the experimental and control groups: respectively, contemporary dance (CD) in full lines and no dance (ND) in dotted lines. Vertical bars are standard errors. Asterisks indicate statistical significant difference (LSD, ^*^P < 0.05,^**^P< 0.01) between pre- and post-test periods within a group.

Despite statistically insignificant third-order interaction, fractal dimension significantly decreased in the CD group in the eyes closed condition (1.69 > 1.66; LSD, *P* < 0.05), whereas a reverse tendency was found in the ND group (1.68 < 1.70; LSD > 0.05), as illustrated in Figure 2A. The pattern was similar in the eyes open condition though *post-hocs* were statistically insignificant (see Figure 2A).

### Diffusion analysis

Results are provided in Table 3. Three-Way ANOVAs with Group, Period and Eye as factors showed only a main effect of Eye for all variables except the critical time interval, which was due to higher values in the eyes closed than in the eyes open condition for the diffusion coefficient *D_S_* [*F*_(1, 36)_ = 24.1, *P* < 0.01; 23.8 > 17.1 mm^2^.s^−1^], the scaling exponent H_S_ [*F*_(1, 36)_ = 5.59, *P* < 0.05; 0.700 > 0.679], and the critical mean square displacement <Δ*r*^2^>_*c*_ [*F*_(1, 36)_ = 25.8, *P* < 0.01; 49.3 > 36.4 mm^2^], and the reverse pattern for the diffusion coefficient *D_L_* [*F*_(1, 36)_ = 6.40, *P* < 0.05; 1.27 < 1.76 mm^2^.s^−1^] and the scaling exponent *H_L_* [*F*_(1, 36)_ = 27.2, *P* < 0.01; 0.050 < 0.088].

**Table 3:** **Mean ± SE of signal diffusion analysis for the groups of participants (CD, contemporary dance; ND, no dance)**.

	**Eyes closed**		**Eyes open**		***F***	***P***
	***CD***	***ND***	***CD***	***ND***		
**D_S_(mm^2^.s^−1^)**						
Pre-test	23.6±2.38	22.8±2.73	17.4±1.82	13.9±1.86		
Post-test	22.5±2.32	26.5±3.74	18.8±1.96	18.1±2.13	<1	–
**D_L_(mm^2^.s^−1^)**						
Pre-test	1.10±0.36	1.14±0.20	2.33±0.56	1.45±0.41		
Post-test	1.65±0.39	1.19±0.44	2.18±0.43	1.10±0.36	<1	–
**H_S_**						
Pre-test	0.699±0.016	0.703±0.013	0.687±0.017	0.655±0.015		
Post-test	0.695±0.018	0.705±0.017	0.699±0.014	0.676±0.012	<1	–
**H_L_**						
Pre-test	0.042±0.013	0.053±0.011	0.100±0.019	0.082±0.017		
Post-test	0.063±0.010	0.042±0.009	0.105±0.016	0.066±0.013	<1	–
**<Δr^2^>_c_ (mm^2^)**						
Pre-test	50.9±5.32	46.0±5.89	37.8±3.64	29.4±3.84		
Post-test	48.9±7.52	51.3±6.48	39.7±5.81	38.6±7.03	<1	–
**Δt_rc_ (s)**						
Pre-test	1.17±0.10	1.08±0.08	1.22±0.12	1.25±0.16		
Post-test	1.12±0.12	1.09±0.11	1.12±0.11	1.09±0.12	<1	–

ANOVAs' F-values are those of the third-order interaction.

### Recurrence quantification analysis

Table 4 shows the detailed results separately for the mediolateral (*x*) and anteroposterior (*y*) planes.

**Table 4:** **Mean ± SE of recurrence quantification analysis for the groups of participants (CD, contemporary dance; ND, no dance)**.

	**Eyes closed**		**Eyes open**		***F***	***P***
	***CD***	***ND***	***CD***	***ND***		
**%REC *x* 2**						
Pre-test	0.464±0.091	0.617±0.192	0.428±0.095	0.480±0.117		
Post-test	0.734±0.159	0.838±0.206	0.607±0.081	0.524±0.104	<1	–
**%REC *x* 3**						
Pre-test	1.36±0.20	1.77±0.47	1.28±0.20	1.44±0.26		
Post-test	1.89±0.32	2.22±0.45	1.70±0.18	1.47±0.23	<1	–
**%DET *x* 2**						
Pre-test	73.6±2.42	74.7±1.72	73.8±1.82	74.9±1.40		
Post-test	76.7±2.19	76.9±1.51	77.2±1.40	75.2±2.10	<1	–
**%DET *x* 3**						
Pre-test	79.2±1.66	76.9±1.52	79.5±1.39	80.2±1.28		
Post-test	81.8±1.64	81.7±1.34	82.1±1.23	80.3±1.47	<1	–
**MAXL *x* 2**						
Pre-test	18.0±4.31	18.0±4.20	15.0±3.11	20.1±6.28		
Post-test	30.6±5.83	25.9±5.45	23.4±3.52	20.9±5.47	<1	–
**MAXL *x* 3**						
Pre-test	43.5±6.88	45.4±6.71	48.0±7.09	49.2±7.51		
Post-test	60.6±8.51	55.3±5.41	54.5±6.83	49.8±7.63	<1	–
**%REC *y* 2**						
Pre-test	0.389±0.085	0.263±0.043	0.394±0.060	0.351±0.080		
Post-test	0.381±0.073	0.311±0.062	0.408±0.88	0.248±0.057	2.51	0.122
**%REC *y* 3**						
Pre-test	1.13±0.21	0.82±0.11	1.12±0.14	1.09±0.21		
Post-test	1.10±0.18	0.95±0.16	1.14±0.19	0.79±0.14	2.80	0.103
**%DET *y* 2**						
Pre-test	73.1±1.51	72.9±1.14	74.5±1.52	72.7±1.18		
Post-test	74.5±1.12	71.1±1.76	74.5±1.30	71.8±0.96	1.49	0.230
**%DET *y* 3**						
Pre-test	75.8±1.83	75.8±1.12	78.0±1.42	76.4±1.32		
Post-test	77.6±1.24	75.0±1.41	78.0±1.29	75.0±0.99	<1	–
**MAXL *y* 2**						
Pre-test	17.6±3.40	11.4±2.35	17.7±2.61	18.7±4.88		
Post-test	18.3±3.78	14.4±3.46	16.6±2.50	8.34±1.56**	4.65	0.038
**MAXL *y* 3**						
Pre-test	38.2±5.69	31.9±4.51	40.8±5.40	38.2±6.61		
Post-test	40.9±6.53	35.2±6.60	36.2±5.49	23.4±3.40	1.53	0.224

ANOVAs' F- and P-values are those of the third-order interaction. Asterisks only indicate statistical significant difference (LSD, P < 0.01) between pre- and post-test periods.

In the mediolateral plane, Three-Way ANOVAs with Group, Period and Eye as factors only showed a main effect of Period for %DET-2% [*F*_(1, 36)_ = 6.72, *P* < 0.05], %DET-3% [*F*_(1, 36)_ = 6.54, *P* < 0.05], MAXL-2% [*F*_(1, 36)_ = 8.19, *P* < 0.01], and MAXL-3% [*F*_(1, 36)_ = 4.83, *P* < 0.05], with higher values in the post- than in the pre-test period: 76.5 > 74.3%, 81.5 > 79.7%, 25.2 > 17.8, and 55.0 > 46.6, respectively.

In the anteroposterior plane, only MAXL was influenced by the factors. Specifically, Three-Way ANOVAs with Group, Period and Eye as factors showed a Period^*^Eye interaction for MAXL-2% [*F*_(1, 36)_ = 8.04, *P* < 0.01], and MAXL-3% [*F*_(1, 36)_ = 8.31, *P* < 0.01] in that, in the eyes open condition, values were lower in the post- than in the pre-test period: 12.4 < 18.2 (LSD, *P* < 0.01), and 29.8 < 39.5 (LSD, *P* < 0.01), respectively.

With respect to our expectation, we observed a Group*Period*Eye interaction for MAXL-2% [*F*_(1, 36)_ = 4.65, *P* < 0.05], which was due to a decrease between the two periods (18.7 > 8.34; LSD, *P* < 0.01) in the ND group in the eyes open condition, as illustrated in Figure 2B.

### Detrended fluctuation analysis

Results are provided in Table 5 for the averaged 21 DFA α exponents in each condition and each plane.

**Table 5:** **Mean ± SE of detrended fluctuation analysis for the groups of participants (CD, contemporary dance; ND, no dance)**.

	**Eyes closed**		**Eyes open**		***F***	***P***
	***CD***	***ND***	***CD***	***ND***		
**DFA** α *x*						
Pre-test	0.961±0.008	0.963±0.006	0.979±0.007	0.924±0.007		
Post-test	1.00±0.010^**^	0.959±0.007	0.987±0.007	0.951±0.006^* *^	39.9	0.000
**DFA** α *y*						
Pre-test	0.835±0.008	0.829±0.006	0.843±0.007	0.780±0.007		
Post-test	0.831±0.009	0.828±0.007	0.846±0.007	0.794±0.006	1.15	0.284

ANOVAs' F- and P-values are those of the third-order interaction. Asterisks only indicate statistical significant difference (LSD, P < 0.01) between pre- and post-test periods.

In both the mediolateral and anteroposterior planes, Three-Way ANOVAs with Group, Period and Eye as factors showed a main effect of Group with higher values in the CD than in the ND group [DFA-α-*x*:0.982 > 0.949, *F*_(1, 796)_ = 14.7, *P* < 0.01; DFA-α-*y*:0.839 > 0.808, *F*_(1, 796)_ = 13.3, *P* < 0.01], a main effect of Eye with higher values in the eyes closed than in the eyes open condition [DFA-α-*x*:0.971 > 0.960; *F*_(1, 796)_ = 8.43, *P* < 0.01; DFA-α-*y*:0.831 > 0.816; *F*_(1, 796)_ = 15.5, *P* < 0.01], and a Group^*^Eye interaction [DFA-α-*x*: *F*_(1, 796)_ = 10.5, *P* < 0.01; DFA-α-*y*: *F*_(1, 796)_ = 48.5, *P* < 0.01] due to higher values, in the ND group, in the eyes closed than in the eyes open condition (DFA-α-*x*:0.961 > 0.937; LSD, *P* < 0.01; DFA-α-*y*:0.829 > 0.787; LSD, *P* < 0.01).

In the mediolateral plane, there was also a main effect of Period [*F*_(1, 796)_ = 31.0, *P* < 0.01] with higher values in the post- than in the pre-test period (0.974 > 0.957). In the anteroposterior plane, there was a Period*Eye interaction [*F*_(1, 796)_ = 5.14, *P* < 0.05] due to a higher value, in the eyes open condition, in the post- than in the pre-test condition (0.820 > 0.812; LSD, *P* < 0.05).

Critical for our hypothesis, there was a Group^*^Period interaction for DFA-α-*x* [*F*_(1, 796)_ = 4.25, *P* < 0.05], due to higher values in the post- than in the pre-test period in both the CD (0.994 > 0.970; LSD, *P* < 0.01) and the ND group (0.955 > 0.943; LSD, *P* < 0.05). Additionally, there was a Group^*^Period^*^Eye interaction for DFA-α-*x* [*F*_(1, 796)_ = 32.9, *P* < 0.01], which was due in the CD group to a higher value in the post- than in the pre-test period in the eyes closed condition (1.00 > 0.961; LSD, *P* < 0.01), whereas in the ND group such an increase was observed in the open eyes condition (0.951 > 0.924; LSD, *P* < 0.01), as illustrated in Figure 2C.

### Counterbalancing effect

Four-Way ANOVA with Order as between-participant factor (two levels: eyes closed then eyes open, and vice-versa), Period (2 levels: pre-test vs. post-test), Eye (2 levels: eyes closed, eyes open) and the measures of posture (26 levels: 6 measures in statistic scores except Romberg quotient, 6 in SDA, 12 in RQA, and 2 in DFA) as within-participant factors showed neither main effect of Order (*F* < 1) nor second-, third-, or forth-order interaction of Order with other factors (*P* > 0.05). Two-Way ANOVA with Order as between-participant factor and Period as within-participant factor showed neither main effect of Order [*F*_(1, 36)_ = 1.18, *P* > 0.05] nor interaction Order^*^Period (*F* < 1) on Romberg quotient.

## Discussion

The present study indicated that older adults trained in CD three times a week for only 1 month modulated their postural control, as revealed in the eyes closed condition by a decrease in fractal dimension and an increase in detrended fluctuation alpha exponent in the mediolateral plane. In contrast, older adults who had not been trained at all showed an increase in length and mean velocity of their postural signal, and in the eyes open condition a decrease in their maximal diagonal line in the anteroposterior plane and an increase in their detrended fluctuation alpha exponent in the mediolateral plane. In the following sections, we start with a comment on methodological aspects of the study, before entering the more substantial discussion of the effects of massed CD training on postural control and that of the putative underlying mechanisms of motor, rather than physical, activity on balance in aging.

In this study, we aimed to investigate the effects of a dance practice, namely CD, on the quality of posture in older adults. Previous studies have pursued this goal using clinical tests such as one-leg stance, functional reach or time up-and-go (e.g., Shigematsu et al., 2002) or clinical batteries such as the Physical Performance Battery (e.g., Hui et al., 2009). A force platform has also been used using static (two- or one-leg stance) and/or dynamic (multidirectional weight shifting) conditions (e.g., Kattenstroth et al., 2013). In the present study, we chose to explore putative CD-induced changes in the quality of posture by measuring the participants' upright stance with their feet oriented in a standard position and in full contact with the platform. Importantly we used dynamic models to explore the CoP fluctuations. Following Collins and DeLuca's seminal work (Collins and De Luca, 1993), our rationale was that static posturography is simpler and safer than dynamic posturography, particularly in older adults, and that dynamic models enable us to gain a “greater understanding of the strategies utilized by the postural control system to maintain the complex, multi-degree-of-freedom structure of the musculoskeletal system in equilibrium with external forces during quiet standing” (Collins and De Luca, 1993, p. 309). In this context, we expected dynamic examination of static posture to make detectable the way CD may have influenced the quality of posture.

To achieve our goal, we invited Bolivian natives to participate in the study, whereas a previous study concerned French natives (Ferrufino et al., 2011). We did not expect intercultural differences to influence the results though future studies may be needed to explore this issue. Participants had no cognitive decline as assessed by the MMSE, to ensure that cognition did not interfere with the quality of their posture (Sheridan and Hausdorff, 2007). Their BMI—26.7(Mean) ± 3.80(*SD*) for all participants—was comparable to that of our previous study, 25.5 ± 4.07 for the whole population (Ferrufino et al., 2011). These values suggested that they may have been overweight on average though normative data are lacking in the present population. Both CD and ND groups had had comparable practice of physical activity in the past, with mostly motor-dominant activity such as gymnastics, dance or martial arts (see below). These observations (BMI, level of activity at baseline) reinforce our view that CD can be practiced at all ages and regardless of physical condition (Ferrufino and Coubard, 2011). Our sample size was 19 in the experimental and control groups, which was comparable to previous intervention studies in this field of research: *N* = 20 vs. 18 for, respectively, the experimental and control groups in Shigematsu et al. (2002), *N* = 14 vs. 12 in Sofianidis et al. (2009), *N* = 14 vs. 14 in Granacher et al. (2012), *N* = 25 vs. 10 in Kattenstroth et al. (2013), *N* = 13 vs. 11 in Krampe (2013). Our age range was 35 years (54–89 years), which was also compatible with previous studies (e.g., range = 32 years in Krampe, 2013), though it could be higher than that of other studies (e.g., range = 19 years in Granacher et al., 2012). Finally, the absence of order effect ruled out our results to be due to this variable.

We now move to the core of the discussion on the effects of CD training on postural control in aging. The first observation is that classical statistic scores (length, surfaces, mean and variance velocities, Romberg quotient) failed to detect any CD-induced changes in postural control, which were only visible in dynamic analyses. Such a contrast had already been reported in our previous study (Ferrufino et al., 2011), which corroborates how useful dynamic models are to identify the subtle modulation of static posture by motor activity. Only length and mean velocity were higher in the ND group between pre- and post-test periods, which may have been due to some degradation of posture that CD may have prevented in the experimental group. Additionally, MAXL decreased in the ND group in the eyes open condition between pre- and post-test periods, whereas it remained stationary in the CD group. How mathematical stability has stabilized in the CD group whereas it has dropped in the ND group remains to be further investigated. Such effect might have been due to the test-retest: participants might have adopted a different strategy to stand upright in the assessment after a period without intervention.

The main result of the present study is that, regardless of the Eye condition, CD tended to decrease fractal dimension whereas a reverse tendency was found in the ND group, resulting in a significant Group*Period interaction. Considering the eyes closed condition, CD significantly decreased fractal dimension whereas a reverse tendency was observed in the ND group. Such a pattern was also observed in the eyes open condition though the decrease in the CD group failed to reach significance. Consistently DFA showed a reverse pattern of results: in the eyes closed condition, CD significantly increased DFA α exponent in the mediolateral plane whereas it remained unchanged in the ND group. In the eyes open condition, CD had no effect on DFA α exponent whereas it increased in the ND group. Indeed fractal dimension is related to DFA hurst exponent (H) following the equation *H* = 2–FD. Thus, fractal dimension inversely coincides with DFA exponent. From a physiological viewpoint, fractal dimension indicates the quantity of exchange between the subject and the environment though it does not inform the quality of exchange—should it be harmony or chaos (Doyle et al., 2005; Hausdorff, 2007). To account for this result, we suggest that CD training reduced the quantity of exchange between the subject and the environment by enabling dancers to make better use of the information available in the environment. In other words, CD trainees, contrary to non-dancers, may have needed less exchange with the world as they use external information in a more precise way. Thus the increase in DFA α exponent may be seen as a higher postural confidence in dancers as compared to non-dancers. Such an effect was more visible in the eyes closed condition and in the mediolateral plane. This could be due to the fact that these conditions are more likely to yield dynamic variations in the postural signal than eyes open (due to lower variability) and anteroposterior plane (due to lower base of support) conditions. As the control group did not undergo any training, it would be premature to assert a motor activity-specific effect, and further investigation is needed to examine whether the observed effects are specific of either CD or other motor training such as fall prevention or Tai Chi Chuan. Our result for fractal dimension is reminiscent with previous studies using fractal measures to capture degrees of human posture complexity, as well as to assess the effects of aging and of interventions on balance (Shimizu et al., 2002; Thurner et al., 2002; Loader et al., 2007). Specifically, Thurner et al. (2002) reported that normal aging is associated with a decrease of postural complexity as assessed by various fractal measures. In other words, non-linear dynamics of central motor control tend to be more regular with age (Thurner et al., 2002), consistent with increased motor stiffness in normal aging as assessed by SDA (Collins et al., 1995). Additionally, Loader et al. (2007) examined the effects of computerized optokinetic therapy (10 sessions over 3 weeks) based on stochastically moving visual stimuli in patients (aged 31–78 years) suffering from unilateral vestibular dysfunction. The authors evidenced that trained patients were improved in several measures of the Sensory Organization Test, as compared to untrained patients. These results and the present study suggest that non-linear dynamic models and measures are useful tools to assess human posture, its aging, and the effects of interventions ought to improve balance.

Taken together with our previous study (Ferrufino et al., 2011), we conclude that a massed practice of CD (12 sessions of 1.5 h each over 1 month only) modulates postural control of older adults in a different way than a distributed practice of CD (20 sessions of 1 h each over 5 months). Massed practice of CD does not influence SDA or RQA but reduces fractal dimension and enhances DFA exponent (the present study), while distributed practice of CD does not influence fractal dimension but enhances the window devoted to stochastic processes and reduces recurrence and mathematical stability of posture (Ferrufino et al., 2011). Such different influences in nature and extent of massed vs. distributed practice of CD does not allow us to conclude about which type of CD training is better than the other but rather suggests that either one or the other should be considered depending upon the strategy pursued by researchers and/or clinicians for the postural rehabilitation of older adults at a given time. It is worth recalling that our CD training was specific in the sense that it trained improvisation, which was at least twofold (Ferrufino and Coubard, 2011). The first type of improvisation, the solo improvisation, practiced in steps (i) and (iv) of the main training stage, enabled trainees to obey their own sensations and the internal logic of kinasesthesia. Such an improvisation could be practiced alone and with eyes closed, explaining why some effects are observed in the eyes closed condition. The second type of improvisation, the framed improvisation, practiced in steps (ii) and (iii) of the main training stage, invited trainees to constantly adapt to constraints (the frame), which were given by a limited space and/or time and/or the presence of other trainees. Such an improvisation was more likely to favor communication and social interaction, should the eyes be closed or open (Ferrufino and Coubard, 2011).

Our studies complement previous intervention studies showing that the practice of dance in aging improves balance as assessed by one-leg stance the eyes closed and functional reach (Shigematsu et al., 2002), dynamic balance as measured by time up-and-go (Hui et al., 2009) or weight shifting in sagittal and frontal planes (Sofianidis et al., 2009), dynamic balance in walking on an instrumental walkway (Granacher et al., 2012), and dynamic balance in multidirectional weight shifting (Kattenstroth et al., 2013). Importantly the same studies have shown that the practice of dance does not improve static balance as assessed by the Physical Performance Battery (Hui et al., 2009) or static posturography (Kattenstroth et al., 2013), unless some difficulty is introduced such as staying one-leg stance on a platform (Sofianidis et al., 2009; Granacher et al., 2012). All studies taken together, we suggest that dance improves dynamic balance as assessed by clinical or laboratory measures, as well as static balance, as soon as either complexity is introduced in the assessment or dynamic analyses of posturography are performed.

We finally move to the mechanisms by which CD may act on postural control. Previous studies have emphasized the need to differentiate between physical-dominant activities, which primarily boost cardiovascular and strength conditions, vs. motor-dominant activities for the motor learning of new skills, which mostly develop balance, fine motor coordination, motor flexibility, and motor speed (Voelcker-Rehage et al., 2010). Following these definitions, walking, jogging, running, cycling, or swimming are physical-dominant activities, whereas dance, gymnastics, yoga, judo, or Tai Chi Chuan belong to motor-dominant activities (Coubard et al., 2011). The former have also been called bioenergetic physical activity and the latter proprioceptive physical activity (e.g., Perrin et al., 2002). Thus dance is primarily a motor-dominant activity, though there is a need to distinguish between different types of dance. Except for aerobic dancing, which has positive effects on cardiovascular health (e.g., Renaud et al., 2010), the results of most intervention studies show dance to be motor-dominant. Indeed Granacher et al. (2012) showed that salsa dance has no effect on leg extensor power, while Kattenstroth et al. (2013) reported that a dance program developed for elderly people did not affect cardio-respiratory health as assessed by spiroergometry (Kattenstroth et al., 2013). In line with these reports, CD is a low-physical impact activity that is therefore open to all regardless of their baseline physical condition (Ferrufino and Coubard, 2011), but high-motor impact activity with beneficial influence on postural control as previously (Ferrufino et al., 2011) and presently evidenced.

In a fMRI study using cognitive tasks, Voelcker-Rehage et al. (2010) showed that high-physically fit participants have higher brain activation in their prefrontal and temporal areas, whereas high-motor fit participants exhibit higher activation in their parietal areas, suggesting that physical vs. motor fitness may counteract aging effects in prefrontal-temporal vs. parietal areas, respectively. Animal studies have evidenced that physical fitness (such as walking on a treadmill or running in a wheel) is associated with angiogenesis in the cerebellum (Black et al., 1990), increased levels of brain-derived neurotrophin factor and of insulin-like growth factor 1, and decreased levels of corticosteroids (Berchtold et al., 2001; Carro et al., 2001; Cotman and Berchtold, 2002), whereas motor fitness (such as acrobatic practice close to fall prevention in humans) is correlated to synaptogenesis and glial hypertrophia in the cerebellum (Black et al., 1990; Anderson et al., 1994). Taken together, these studies suggest that physical- vs. motor-dominant activities have different effects on the central nervous system (CNS) at both cell and macroscopic scales. Motor control involves several areas of the CNS from the brainstem (vestibular nuclei, reticular formation, cerebellum) to subcortical (globus pallidus, putamen, caudate nucleus) and cortical areas (supplementary motor area, frontal eye field, primary motor cortex). To account for the positive effects of CD on attention (Coubard et al., 2011) and posture (Ferrufino et al., 2011), we have suggested that dance may stimulate cortical-subcortical loops, particularly the dorsolateral prefrontal loop bridging the dorsolateral prefrontal cortex to striatum, pallidum, substancia nigra, and thalamus (Alexander et al., 1986). Indeed, the high attentional (Coubard et al., 2011) and motor (Ferrufino et al., 2011) flexibility required for improvisation may play a role in boosting such cortical-subcortical circuitry. Additionally, the fact that improvisation can be trained with participants' eyes closed in the solo improvisation is compatible with a recruitment not only of the parvocellular visual pathway but also of the magnocellular one, which itself projects to both subcortical and cortical areas (Bullier, 2001), explaining why some beneficial effects may be observed in the eyes closed condition.

To conclude, this intervention showed that practice of CD for only 1 month had beneficial impact on postural control in older adults. Such a benefit took the form of lower exchange between the subject and the environment, which we interpreted as a higher postural confidence in dancers compared to non-dancers. Massed practice of CD thus has different effects in nature and extent than distributed practice of CD over several months. Since CD has beneficial effects on attention and posture, and may be practiced with or without vision and individually or in interaction with other trainees, it should be recommended in adults regardless of age, physical, cognitive, and social conditions in order to optimize successful aging (Hank, 2011).

### Conflict of interest statement

The authors declare that the research was conducted in the absence of any commercial or financial relationships that could be construed as a potential conflict of interest.
